# Risky Decision Making Under Stressful Conditions: Men and Women With Smaller Cortisol Elevations Make Riskier Social and Economic Decisions

**DOI:** 10.3389/fpsyg.2022.810031

**Published:** 2022-02-04

**Authors:** Anna J. Dreyer, Dale Stephen, Robyn Human, Tarah L. Swanepoel, Leanne Adams, Aimee O'Neill, W. Jake Jacobs, Kevin G. F. Thomas

**Affiliations:** ^1^ACSENT Laboratory, Department of Psychology, University of Cape Town, Cape Town, South Africa; ^2^Clinical and Experimental Sciences, Faculty of Medicine, University of Southampton, Southampton, United Kingdom; ^3^Anxiety Research Group, Department of Psychology, University of Arizona, Tucson, AZ, United States

**Keywords:** cortisol, decision-making, sex differences, stress, chatroom

## Abstract

Men often make riskier decisions than women across a wide range of real-life behaviors. Whether this sex difference is accentuated, diminished, or stable under stressful conditions is, however, contested in the scientific literature. A critical blind spot lies amid this contestation: Most studies use standardized, laboratory-based, cognitive measures of decision making rather than complex real-life social simulation tasks to assess risk-related behavior. To address this blind spot, we investigated the effects of acute psychosocial stress on risk decision making in men and women (*N* = 80) using a standardized cognitive measure (the Iowa Gambling Task; IGT) and a novel task that simulated a real-life social situation (an online chatroom in which participants interacted with other men and women in sexually suggestive scenarios). Participants were exposed to either an acute psychosocial stressor or an equivalent control condition. Stressor-exposed participants were further characterized as high- or low-cortisol responders. Results confirmed that the experimental manipulation was effective. On the IGT, participants characterized as low-cortisol responders (as well as those in the Non-Stress group) made significantly riskier decisions than those characterized as high-cortisol responders. Similarly, in the online chatroom, participants characterized as low-cortisol responders (but not those characterized as high-cortisol responders) were, relative to those in the Non-Stress group, significantly more likely to make risky decisions. Together, these results suggest that at lower levels of cortisol both men and women tend to make riskier decisions in both economic and social spheres.

## Introduction

Men tend to make riskier decisions than women across a wide range of real-life situations (Byrnes et al., 1999; d'Acremont and Van der Linden, 2006; Charness and Gneezy, 2012; Georgiou et al., 2018; Sidlauskaite et al., 2018). Whether stressful conditions accentuate, diminish, or have no effect on this sex difference is in dispute. Some studies report that, after exposure to laboratory-induced acute psychosocial stressors, men tend to make risky decisions whereas women tend to make safer, more risk-averse decisions (Preston et al., 2007; Lighthall et al., 2009, 2012; van den Bos et al., 2009, 2014; Mather and Lighthall, 2012; Daughters et al., 2013; Alacreu-Crespo et al., 2019). Other studies suggest that, after exposure to laboratory-induced acute psychosocial stressors, men (Fairchild et al., 2009), but not women (Cahlíková and Cingl, 2017), tend to make safer, more risk-averse decisions. A third set of studies reports no such sex differences (Starcke et al., 2008; Gathmann et al., 2014), with some reporting that, universally, stressor-exposed individuals are either more risk averse than their unexposed counterparts (Fairchild et al., 2009; Mather et al., 2009; Clark et al., 2012; Schwabe et al., 2013) or no different at all in their approach to economic decision-making tasks (Sokol-Hessner et al., 2016).

Starcke and Brand's (2016) meta-analysis of 32 studies that used a laboratory-based stress induction method and that measured a decision-making outcome (cumulative *N* = 1,829) found that (a) stress-exposed individuals are more likely to make risky decisions, and (b) sex is not a significant moderator of the relationship. The authors noted that differences in study designs predict the magnitude of the effect. For example, stress exposure increased risky decision making in task situations where risk taking was disadvantageous (i.e., when taking a riskier decision led to fewer rewards; *d* = 0.26) but did not do so in other task situations (*d* = 0.01). Similarly, studies using inductions such as the Trier Social Stress Test (TSST; Kirschbaum et al., 1993), where stress results from appraisal and cognitive processing of a social situation that is not an immediate physical threat (*processive stressors*), reported significant increases in risky decision making (*d* = 0.19), whereas those using inductions such as the Cold Pressor Test (CPT; Hines and Brown, 1936), where stress results primarily from experiences of physiological stress, did not (*d* = 0.09).

Of note here is that in studies using processive stressors the moderating effect of sex almost reached the threshold for statistical significance (*p* = 0.051), with men showing increases and women showing decreases in risky decision making.

Starcke and Brand's meta-analysis did not consider differences in the ecological and/or face validity of decision-making tasks. Hence, it remains possible that results from traditional laboratory-based tasks do not generalize to decision making in social contexts (Bruine de Bruin et al., 2007; Shields et al., 2016). It is also possible that relations among sex, risky decision making, and stress will be different under laboratory vs. real-life conditions. For example, Starcke et al. (2016) used the Waste Water Treatment Simulation (a computerized decision-making scenario with high face validity that offers participants high-risk, high-reward options vs. safer, lower-reward options) and found that, in direct contrast to the meta-analytic results, TSST exposure increased risky decision making in women but not in men. Therefore, research investigating risky decision making in scenarios that simulate real-word situations, especially social contexts, appears to be a valuable endeavor.

### The Current Study

Our primary interest was in the effects of acute psychosocial stress on risky decision making by healthy young men and women in a social context that simulated a real-life situation (an online chatroom). Exposure to acute psychosocial stress activates the hypothalamic-pituitary-adrenal axis response that results in release of cortisol from the adrenal cortex (Alderson and Novack, 2002; Dickerson and Kemeny, 2004; Wolf, 2019). Because cortisol is one of the physiological elements responsible for the effects of stress on decision making (Putman et al., 2010; Pabst et al., 2013a,b), in this study we used elevated cortisol levels as a marker of the stress response.

To increase the sensitivity and generalizability of our results, we used (a) a laboratory-based decision-making task where risk taking was disadvantageous (i.e., the Iowa Gambling Task [IGT]; Bechara et al., 1994), and (b) a standardized stress-induction method that featured strong elements of a processive stressor. Additionally, we used a novel task that simulated a real-life social situation (an online chatroom where we analyzed the participant's willingness to meet another user in person) to measure the effects of stress on decision making. This task was embedded seamlessly in the study protocol, allowing us to retain naturalistic observation within standardized, controlled experimental conditions.

Online chatrooms have become an increasingly popular way to meet people and to find prospective sexual partners (Walker and Bakopoulos, 2005; Chou and Peng, 2007; Swanepoel and Thomas, 2012). Empirical studies suggest that chatroom scenarios are reliable methods that allow investigation of real-world social interactions under strictly controlled conditions (Subrahmanyam et al., 2006; Silk et al., 2011). To our knowledge, no published study has used chatroom scenarios to manipulate and observe risk decision making.

We conducted an analysis that separated stressor-exposed participants into two sub-groups, one comprised of high-cortisol responders (i.e., those with large cortisol elevations after stressor exposure) and the other of low-cortisol responders (i.e., those with small cortisol elevations after stressor exposure). Using this strategy, van den Bos et al. (2009) showed that post-stressor female (but not male) IGT performance followed an inverted *U*-shaped curve (i.e., safer decision making occurred at moderate levels of cortisol elevation whereas risky decision making occurred at very low or very high concentrations).

In summary, we used healthy men and women as participants, a standardized laboratory-based measure (the IGT) where risk taking is disadvantageous, and a novel social simulation (an online chatroom) where risk taking is, ultimately, potentially advantageous, to test three specific hypotheses based on the existing literature: (1) individuals experiencing stress-related cortisol release make more risky decisions than stressor-unexposed individuals; (2) men experiencing stress-related cortisol release show more risk-taking behavior than women experiencing stress-related cortisol release; and (3) in women experiencing stress-related cortisol release, those with a relatively low magnitude of cortisol elevation make safer decisions than those experiencing a relatively high magnitude of elevation, who make riskier decisions.

## Methods

### Participants

We used convenience sampling (i.e., advertisements on the website of our department's student research subject pool and on our university's intranet) to recruit 80 healthy undergraduate students (40 women and 40 men), aged 18–25 years (*M* = 20.11 ± 1.46). We then used stratified random assignment to constitute four study groups: Female Stress, Female Non-Stress, Male Stress, and Male Non-Stress (*n* = 20 each).

The sampling method differed for men and women. This difference arose because it was important to determine female participants' menstrual cycle stage and to exclude women who were taking oral contraception. The use of oral contraceptives and the phase of the menstrual cycle both affect the stress response because they influence endogenous cortisol levels (Kudielka and Kirschbaum, 2005; Kuhlmann and Wolf, 2005). Hence, men who wished to participate signed up on the subject pool website or by responding to the advertising email. In contrast, women who wished to participate were asked to email the researcher to determine which day would be appropriate for testing, and to confirm that they were not taking oral contraceptive medication. This procedure ensured confidentiality of potentially sensitive information. It is best to test female participants in the luteal phase of their menstrual cycle (i.e., the 12 days preceding the start of their menses); research indicates that women in this phase of their menstrual cycle experience similar baseline levels of cortisol to men (Kirschbaum et al., 1999). Interested women were also asked, *via* email, to estimate when their next menses would start. Using this information, the researcher scheduled a testing date for when she would be in the luteal stage of her menstrual cycle. At the conclusion of the experimental procedures, each female participant was asked to email the researcher on the first day of her next period to ensure she was tested during the luteal phase. Data from those not in the luteal phase, and from those who appeared to be experiencing irregular menstrual cycles, were excluded from the study.

General exclusion criteria were (a) smoking cigarettes regularly, (b) a Beck Depression Inventory-Second Edition (BDI-II; Beck et al., 1996) score ≥ 29 (severe depression), (c) current use of psychoactive or steroid-based medication, or (d) a body mass index (BMI) of < 19 (underweight) or > 31 (obese). These factors are potentially confounding variables in research investigating the effects of psychosocial stress on cognitive performance and are consistent with criteria used in previous research (Kudielka et al., 2009; Herhaus and Petrowski, 2018; Miller et al., 2018). Participants were asked to refrain from eating or drinking anything (except water), and from taking part in any form of strenuous exercise, for at least 2 h before their test session.

Each participant received either course credit or monetary compensation (the equivalent of ~US$3.25) in exchange for their involvement in the study. The relevant research ethics committees at our institution approved the study protocol. All participants gave written informed consent in accordance with the Declaration of Helsinki (Association, 2013).

### Materials and Procedure

**Figure 1:**
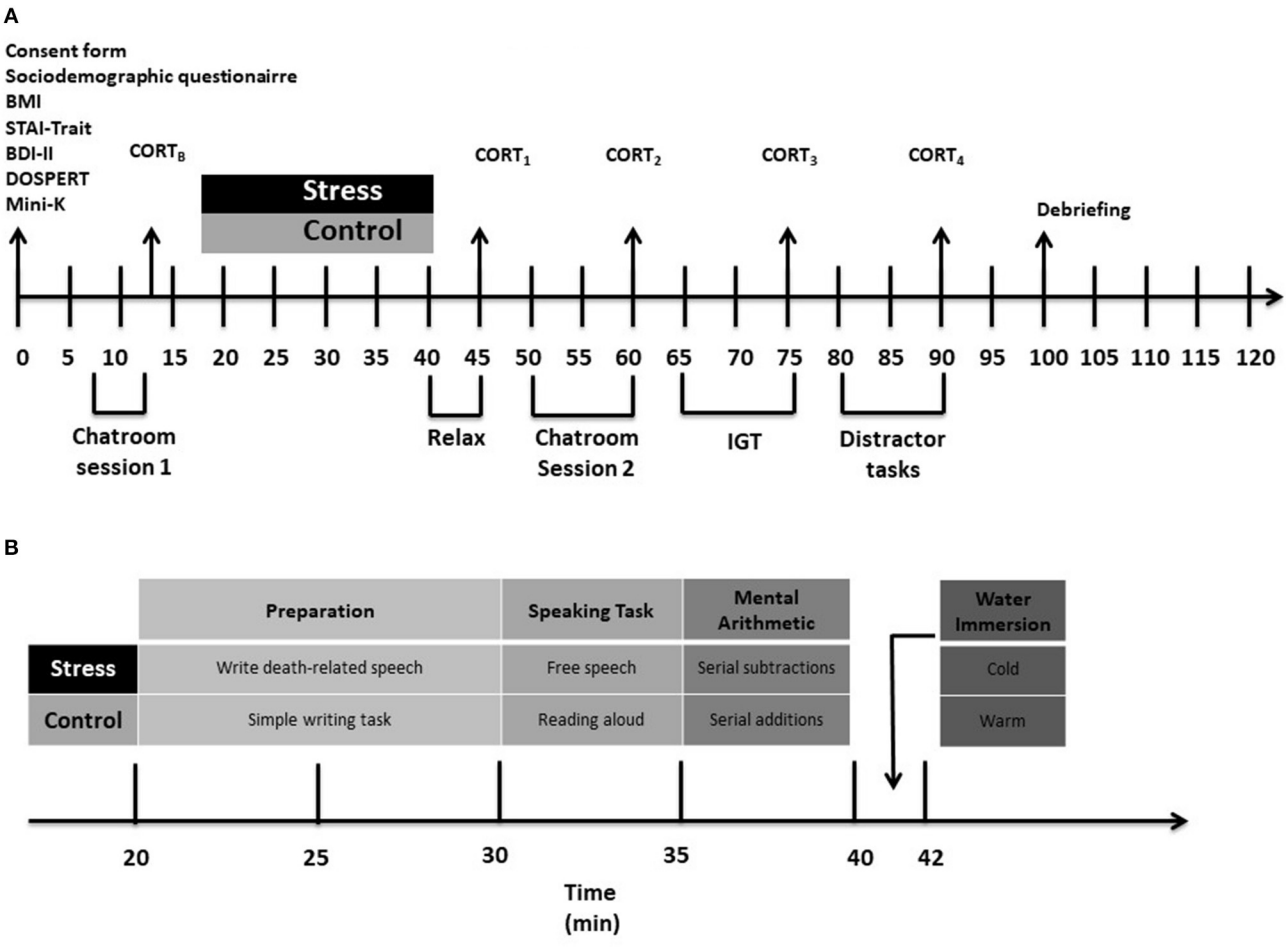
A flowchart describing the study procedure. **(A)** Shows the order of the measures taken throughout the study. **(B)** Shows the procedure followed for the experimental manipulation. CORT_B_, baseline cortisol measurement [in the notation favored by, for instance, Smeets et al. (2012), this is *t*-27 because it is taken 27 mins before stressor offset, which is noted as *t* + 00]; CORT_1_, 2nd cortisol measurement (*t* + 5); CORT_2_, 3rd cortisol measurement (*t* + 20); CORT_3_, 4th cortisol measurement (*t* + 35); CORT_4_, 5th cortisol measurement (*t* + 50). STAI_B_, Baseline state anxiety measurement; STAI1, 2nd state anxiety measurement; STAI_2_, 3rd state anxiety measurement; STAI3, 4th state anxiety measurement; STAI_4_, 5th state anxiety measurement. BMI, body mass index; STAI, State-Trait Anxiety Inventory; BDI-II, Beck Depression Inventory-Second Edition; DOSPERT, A Domain-specific Risk-taking Scale; MSST, Mortality Salience Stress Test; IGT, Iowa Gambling Task.

Figure 1 presents a flowchart describing the experimental procedures.

Procedures for each participant were administered individually, within a single 2-h session, in a university research laboratory. Each session was run between 14h00 and 18h00 to control for cortisol's diurnal cycle. Cortisol has a circadian rhythm, with levels peaking in the morning just after waking, and decreasing slowly over the course of the day, with the lowest levels in the late afternoon and evening (Maheu et al., 2005; Seddon et al., 2020). Studies using acute psychosocial stressors should run in the late afternoon, when cortisol levels are at their lowest and most constant, as this is when cortisol changes due to a stressor will be most easily identified (Dickerson and Kemeny, 2004; Kudielka et al., 2009).

Upon arrival at the laboratory, a research assistant (RA) greeted the participant. The RA asked the participant to read and sign an informed consent document and to then complete a study-specific sociodemographic questionnaire that gathered information about the participant's age, sex, medical and psychiatric history, use of chronic and/or steroidal medications, and smoking behavior. Then, to facilitate BMI calculation, the RA measured the participant's height and weight. The participant then completed the BDI-II, the State-Trait Anxiety Inventory-Trait Form (STAI-Trait; Spielberger et al., 1983), the Domain-Specific Risk-Taking (DOSPERT) Scale (Blais and Weber, 2006), and the Mini-K questionnaire (Figueredo et al., 2006). The DOSPERT and Mini-K gathered information about, respectively, the participant's risk-taking tendencies or intentions and his/her Life History Strategy (Figueredo et al., 2007).

After the participant's eligibility had been established by perusing data from the sociodemographic questionnaire, BDI-II, and BMI calculation, the researcher then instructed them to “kill time” in an online chatroom while the RA prepared the experimental procedures.

We designed this chatroom to facilitate observation of behavior that might indicate risky decision making in a real-life social situation. We developed standardized chatroom scripts and loaded them into the chatroom interface so that when the participant entered the room it appeared that conversation was ongoing for some time. Three confederates, each of whom played a specific role, were logged into the chatroom before the participant entered. For male participants, the characters present in chatroom were Commgirl (female), 2_cool (female), and JP13 (male); for female participants, they were ThomasTheTank Engine (male), Packetman (male), and Coolgirl (female; see Supplementary Material for detailed character descriptions). The purpose of this pre-manipulation time in the chatroom, which we designated as Chatroom Session 1, was to familiarize the participant with the nature and operation of the interface and with the personalities of the other users (i.e., the three confederates).

After 5 mins in the chatroom, the experimental manipulation took place. Those assigned to the Non-Stress condition were exposed to a control condition that mirrored elements of the acute psychosocial stressor. First, the RA asked the participant to write a summary of everything they had done that day. After 10 mins of that activity, the RA escorted the participant to an adjacent room. This room contained no equipment and no other people. The RA asked the participant to stand and read aloud from a general interest magazine for 5 mins, to then count upwards in multiples of 5 for 5 mins, and, finally, to submerge the dominant arm in warm water (34–38°C) for up to 2 mins.

Those assigned to the Stress condition were exposed to a stress-induction procedure (Adams and Minnozzi, 2013; Du Plooy et al., 2014) that, because it combined elements of the TSST and CPT in attempting to increase cortisol elevations in response to stress, is similar to the Maastricht Acute Stress Test (MAST; Smeets et al., 2012). First, the RA asked the participant to prepare a 5-min speech describing the circumstances of their own death in detail (instructions patterned after Landau et al., 2004, p. 427). The participant was given 10 mins to prepare. The RA then escorted the participant to an adjacent room.

There, while under the spotlight of a halogen lamp, the participant was instructed to deliver the speech extemporaneously while looking directly into the lens of a video camera and being observed by two judges. After the 5-min speech, the judges instructed the participant to complete a mental arithmetic task (serially subtract 17, starting from 2,043, continuously for 5 mins; if the participant made an incorrect subtraction, the judges instructed them to start again at 2,043) and to then submerge the dominant arm in cold water (0–4°C) for up to 2 mins.

After the experimental manipulation, the participants in both the Non-Stress and Stress conditions relaxed for 5 mins. The researcher then asked each participant to again “kill time” in the chatroom. This time, the tone of conversation in the room was more animated and sexual. To set the tone, the confederates discussed their willingness to take someone home after meeting them for the first time at a party. Hence, the purpose of this post-manipulation time in the chatroom, which we designated as Chatroom Session 2, was to offer the participant the opportunity to display risky decision-making behavior in the online (and seemingly real-life) chatroom. Immediately after the participant exited the chatroom, the RA administered a standard computerized version of the IGT.

#### Salivary Cortisol Measures

To assess the magnitude of the physiological stress response provoked by the experimental manipulation we used SARSTEDT Salivette® cortisol swabs (Sarstedt, Nümbrecht, Germany) to take saliva samples at five points during the study protocol. The first, a baseline measure (CORT_B_) was taken before the participant underwent the experimental manipulation. The second (CORT_1_) was taken after the 5-min rest period that followed the experimental manipulation, the third (CORT_2_) immediately after Chatroom Session 2 (i.e., ~20 mins following stressor offset, at the point when peak magnitudes of cortisol elevation are generally expected; Dickerson and Kemeny, 2004), the fourth (CORT_3_) immediately after the IGT (i.e., ~35 mins following stressor offset), and the fifth (CORT_4_) 15 mins after that.

At each measurement point, the participant was required to chew a swab for 1 min. This is an easy, effective, non-intrusive, and non-stressful way to collect cortisol samples (Garde and Hansen, 2005). After the samples were collected, they were stored immediately in individual, labeled tubes, and were frozen until transportation to a local hospital laboratory for analysis. They were analyzed using a competitive electrochemiluminescent immunoassay on the Roche Cobas 6000 (Roche Diagnostics GmbH, Mannheim, Germany) with a coefficient of variation of 4%.

#### Measures of Risk Decision Making

##### Iowa Gambling Task

The IGT (Bechara, 2007) is one of the most used laboratory measures of risky decision making. It requires participants to develop a profitable monetary strategy by selecting cards from four piles. Each card indicates a monetary gain and a possible penalty. In this task, there is a conflict between the probability of gaining an immediate large reward in two long-term disadvantageous decks of cards (A and B) and the probability of gaining an immediate small reward in two long-term advantageous decks of cards (C and D). Risky decision making in this task is indicated by the continuous selection of cards from high win-high loss piles (A and B), which over time results in an overall unsuccessful outcome.

##### Online Chatroom

We recorded two types of behavior in the online chatroom: A participant's unprompted offer to meet one of the chatroom confederates in real life (this offer could occur at any time in the chatroom) or their response to an offer to meet in real life (one of the confederates made such an offer 5 mins into the Chatroom Session 2). We also recorded how much detail the participant offered or requested regarding the meeting (e.g., a contact number, a meeting place or time). Therefore, risky decision making in this task is indicated by deciding to meet, and/or providing personal information to, someone whose acquaintance has only been made in the chatroom. Such a meeting, even in a public place and even given that participants are told explicitly that other people in the chatroom are first-year undergraduate students, like them, is a short-term (proximate) risk fraught with potential hazards (i.e., the possibility of social rejection). Despite the proximate risk, we argue that making or accepting an offer to meet someone from the chatroom is an advantageous risk-taking behavior (as it would be in other real-life online chatrooms) because a basic human desire as social beings is to broaden one's social circle and/or find a romantic partner. The chatroom offers the potential for long-term (ultimate) reward; the greater the risk taken (by offering specific details of the meeting), the greater the chance of a potential reward (making a new friend or finding a romantic partner). These ultimate rewards outweigh the proximate risks; this is a contingency trap as defined in the operant literature (see Figueredo and Jacobs, 2010; Baum, 2016), and hence proposing or accepting the meeting is advantageous^1^.

### Data Management and Statistical Analyses

We used RStudio Version 0.98.1028 to complete all analyses, with the threshold for statistical significance set at α = 0.05. For all ANOVAs described below, Type I Sums of Squares was used and the order in which the variables are described is the order in which the variables were placed in the model. The analytic plan proceeded across four discrete steps.

First, 2 × 2 (Experimental Condition [Stress/Non-Stress] × Sex [male/female]) factorial ANOVAs assessed sample characteristics (age, BMI, scores on the BDI-II, STAI-Trait, DOSPERT Scale, and Mini-K) to determine if all participants, regardless of group assignment, were drawn from the same population. Ensuring there are no between-group differences with respect to these variables is important because they have independent effects on cortisol levels and/or tendency toward risky decision making.

Second, a 2 × 2 × 4 repeated-measures ANOVA (Experimental Condition [Stress/Non-Stress] × Sex [male/female] × Measurement Point [T1/T2/T3/T4]) for each of five difference-score outcome variables (CORTΔ1/CORTΔ2/CORTΔ3/CORTΔ4)^2^ assessed the effectiveness of the experimental manipulation.

Third, we characterized (separately, for men and women) participants in the Stress condition as low-cortisol responders (LowCort) or high-cortisol responders (HighCort). To do this, we subtracted, for each participant in the Male Stress and Female Stress groups, the baseline cortisol value (CORT_B_) from the value at the measurement point where we expected the peak cortisol responses (CORT_2_), thus deriving the CORTΔ2 variable. We then used a median split of these difference scores for men and women separately to create sex-specific LowCort and HighCort groups. Thereafter, we again assessed the effectiveness of the experimental manipulation by conducting, for the male and female data separately, a series of 3 (Group: Non-Stress, LowCort, HighCort) × 5 (Measurement Point: CORT_B_, CORT_1_, CORT_2_, CORT_3_, CORT_4_) repeated-measures ANOVAs. We applied log transformations where necessary to improve normality of the data distribution.

Fourth, we analyzed two outcome variables for the IGT data, each measured over 5 blocks of 20 trials each: (a) CD-AB Cards score and (b) cumulative money earned. To derive the *CD-AB Cards* outcome variable, we subtracted the total number of AB cards selected from the total number of CD cards selected in each 20-trial block. The selection of more AB than CD cards is an indication of risky decision making (Preston et al., 2007; Lighthall et al., 2012), and hence the lower the CD-AB score, the greater the risk decision making. To derive the *cumulative money earned* outcome variable, we considered that participants start each block with a loan of €2,000—values higher than that indicate net wins whereas values lower than that indicate net losses. Safe decision-making results in more money earned. For each outcome variable, we conducted a 3 (Group: Non-Stress, LowCort, HighCort) × 5 (Block: IGT_1_, IGT_2_, IGT_3_, IGT_4_, IGT_5_) × 2 (Sex: Women vs. Men) repeated-measures ANOVA. Note that for this analysis, and for the one described below, we collapsed male and female data so there was only one Non-Stress, one LowCort, and one HighCort group.

Finally, we analyzed data from the online chatroom regarding each participant's (a) offer to meet another chatroom user in person or (b) response to the confederate's offer to meet in person. The participant's behavior was coded on a 4-point ordinal scale: 3 = participant made an unprompted offer to another user and gave details of where to meet; 2 = participant accepted an offer to meet and gave details; 1 = participant accepted or made an offer to meet and gave limited details; or 0 = participant rejected the offer. This variable, *willingness to take a risk*, captured the participant's relative willingness to meet someone from the chatroom in real-life (a proxy for risk-taking in real-world circumstances). An ordinal logistic regression model examined whether group membership (HighCort, LowCort, Non-Stress) and Sex (female, male) predicted willingness to take a risk.

## Results

### Sample Characteristics

Analyses of the Table 1 data detected no statistically significant main effects of Experimental Condition or Sex, and no statistically significant interaction effect, on participant age or BMI, or on BDI-II, STAI-Trait, DOSPERT Scale, or Mini-K scores, *F*s < 3.30, *p*s >0.075.

**Table 1:** Sample characteristics: Descriptive statistics (*N* = 80).

	**Group**			
	**Stress**		**Non-Stress**	
	**Male**	**Female**	**Male**	**Female**
**Measure**	**(*n* = 20)**	**(*n* = 20)**	**(*n* = 20)**	**(*n* = 20)**
Age	20.25 (0.91)	19.60 (1.43)	20.30 (1.72)	20.30 (1.63)
BDI-II	7.60 (6.56)	11.50 (6.59)	10.25 (7.89)	10.90 (6.90)
STAI—Trait	38.55 (10.87)	42.70 (11.67)	39.95 (11.02)	39.40 (10.77)
BMI	23.17 (2.68)	23.41 (3.44)	23.51 (3.15)	23.32 (2.86)
Mini-K	22.84 (10.90)^a^	30.25 (7.12)	23.45 (13.16)	25.10 (12.12)
DOSPERT	108.22 (20.92)^b^	94.75 (16.54)^c^	96.22 (21.05)^c^	96.64 (21.51)^d^

*Means are provided with standard deviations in parentheses. BDI-II, Beck Depression Inventory-Second Edition; STAI, State-Trait Anxiety Inventory; BMI, body mass index; DOSPERT, Domain-Specific Risk-Taking Scale*.

a *n = 19*.

b *n = 12*.

c *n = 9*.

d *n = 11*.

### Manipulation Check

Data for one participant in the Female Stress group were excluded from all analyses because her cortisol levels were much higher than the rest of the sample. For instance, her baseline cortisol level (394.10 nmol/l) was 134.40 *SD* above the mean of the Female Stress group (*M* = 7.03 ± 2.88), and her final (Time 4) cortisol sample (267.10 nmol/l) was 94.77 *SD* above that group mean (*M* = 7.43 ± 2.74). Her cortisol readings at Time 2, Time 3, and Time 4 were 49.68 nmol/l, 60.01 nmol/l, 79.27 nmol/l, respectively. There may have been errors in data collection and/or cortisol assay for this participant's saliva samples.

Absolute cortisol values at baseline were significantly different for participants in the Male Stress and Female Stress groups, *p* = 0.034. This result supports our decision to focus on difference-score outcome variables (i.e., CORTΔ1/CORTΔ2/CORTΔ3/CORTΔ4) when analyzing the cortisol data.

Factorial ANOVA did not detect a significant main effect of Sex, *F*_(1, 300)_ = 1.68, *p* = 0.196, $\eta_{\text{p}}^{2}$ = 0.006, but did detect a significant main effect of Experimental Condition, *F*_(1, 300)_ = 126.93, *p* <0.001, $\eta_{\text{p}}^{2}$ = 0.297, and of Measurement Point, *F*_(3, 300)_ = 4.58, *p* = 0.004, $\eta_{\text{p}}^{2}$ = 0.044, a significant Experimental Condition × Sex interaction, *F*_(1, 300)_ = 16.72, *p* < 0.001, $\eta_{\text{p}}^{2}$ = 0.053, and a significant Experimental Condition × Measurement Point interaction, *F*_(3, 300)_ = 3.48, *p* = 0.016, $\eta_{\text{p}}^{2}$ = 0.033. It did not detect a significant Measurement Point × Sex interaction, *F*_(3, 300)_ = 0.58, *p* = 0.629, $\eta_{\text{p}}^{2}$ = 0.006, or a significant three-way interaction, *F*_(3, 300)_ = 0.86, *p* = 0.460, $\eta_{\text{p}}^{2}$ = 0.009.

*Post-hoc* pairwise comparisons using Tukey's HSD test, which examined the significant Experimental Condition × Sex interaction, found that overall and on average across the four measurement points, (a) Male Stress participants exhibited significantly greater increases in salivary cortisol levels than Female Stress participants, *p* < 0.001, and (b) salivary cortisol elevations did not differ significantly across the Male and Female Non-Stress groups, *p* = 0.209.

Similar analyses examining the significant Experimental Condition × Measurement Point interaction found that overall and on average across men and women, participants in the Stress groups exhibited a significantly greater salivary cortisol increase than those in the Non-Stress groups, *p*s < 0.028.

These results indicate that, at each measurement point, the magnitude of cortisol increase over baseline was greater for stressor-exposed than for non-exposed participants. Moreover, within the group of stressor-exposed participants, men experienced markedly higher elevations of cortisol post-manipulation than women. In contrast, unexposed men and women showed similar (relatively low) levels of cortisol post-manipulation. Because this sex difference in magnitude of cortisol reactivity could have affected subsequent statistical analyses, we separated the data from the Male and Female Stress groups, did a median split for each group, and assigned the participants' data to Male HighCort, Male LowCort, Female HighCort, and Female LowCort groups.

The categorization of groups in this way is supported by linear regression analyses indicating that a continuous cortisol variable (CORTΔ2) is a significant predictor of risky decision making (please see the Supplementary Material file for a summary of these analyses).

#### Men

**Figure 2:**
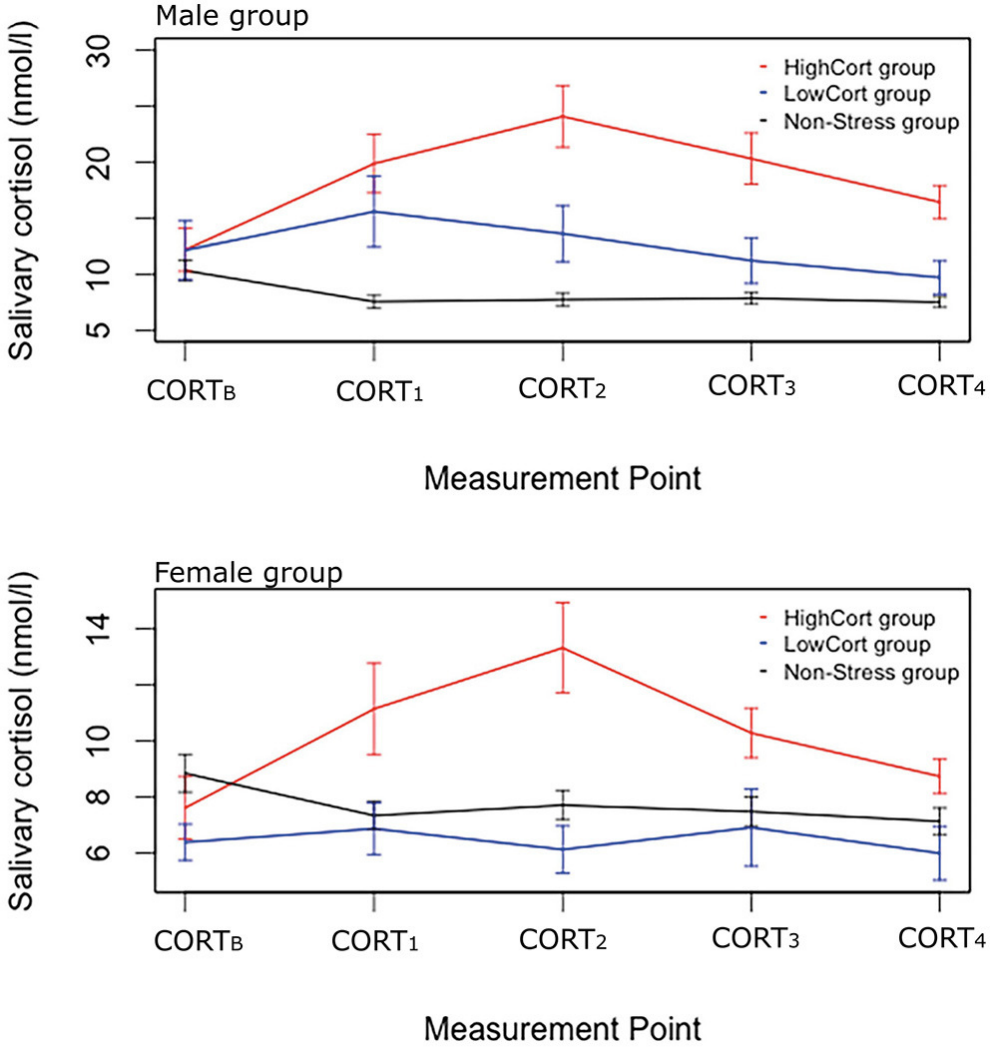
Salivary cortisol levels across the experimental session for the three sub-groups. Male data are in the upper panel, and female data in the lower panel. Error bars indicate standard error of means, with 95% confidence interval.

The median split assigned 10 men to the HighCort group and 10 to the LowCort group (LowCort < 4.875 nmol/l < HighCort). Analysis of the log-transformed data detected a significant main effect of Group, *F*_(2, 185)_ = 68.13, *p* < 0.001, $\eta_{\text{p}}^{2}$ = 0.42, but not of Measurement Point, *F*_(4, 185)_ = 1.09, *p* = 0.364, $\eta_{\text{p}}^{2}$ = 0.02, and a significant Group × Measurement Point interaction, *F*_(8, 185)_ = 3.54, *p* <0.001, $\eta_{\text{p}}^{2}$ = 0.13. Follow-up pairwise comparisons using Tukey's HSD test indicated that, on average, (a) participants in the Male HighCort, Male LowCort, and Male Non-Stress groups had similar cortisol levels at baseline, *p*s >0.99, (b) those in the Male HighCort group had significantly higher levels than those in the Male Non-Stress group at CORT_B_, CORT_1_, CORT_2_, and CORT_3_, *ps* <0.001, (c) those in the Male HighCort group had significantly higher levels than those in the Male LowCort group at CORT_2_, *p* = 0.016, and at CORT_3_, *p* = 0.014, and (d) those in the Male LowCort group had significantly higher levels than those in the Male Non-Stress group at CORT_1_, *p* = 0.009 (see Figure 2, upper panel).

#### Women

The median split assigned 10 women to the HighCort group and 9 to the LowCort group (LowCort < 1.65 nmol/l < High Cort). Analysis of the log-transformed data detected a significant main effect of Group, *F*_(2, 180)_ = 21.05, *p* <0.001, $\eta_{\text{p}}^{2}$ = 0.19, but not of Measurement Point, *F*_(4, 180)_ = 0.87, *p* = 0.486, $\eta_{\text{p}}^{2}$ = 0.02, and a significant Group × Measurement Point interaction, *F*_(8, 180)_ = 2.07, *p* = 0.041, $\eta_{\text{p}}^{2}$ = 0.08. Follow-up pairwise comparisons, similar to those described above, indicated that participants in the Female HighCort group had significantly higher salivary cortisol levels than those in the other two groups (vs. Non-Stress, *p* = 0.014; vs. LowCort, *p* <0.001) at CORT_2_. Analyses detected no other significant between-group differences at any of the other measurement points (see Figure 2, lower panel).

### Performance on Decision-Making Tasks

#### Risky Decision Making on the IGT

**Figure 3:**
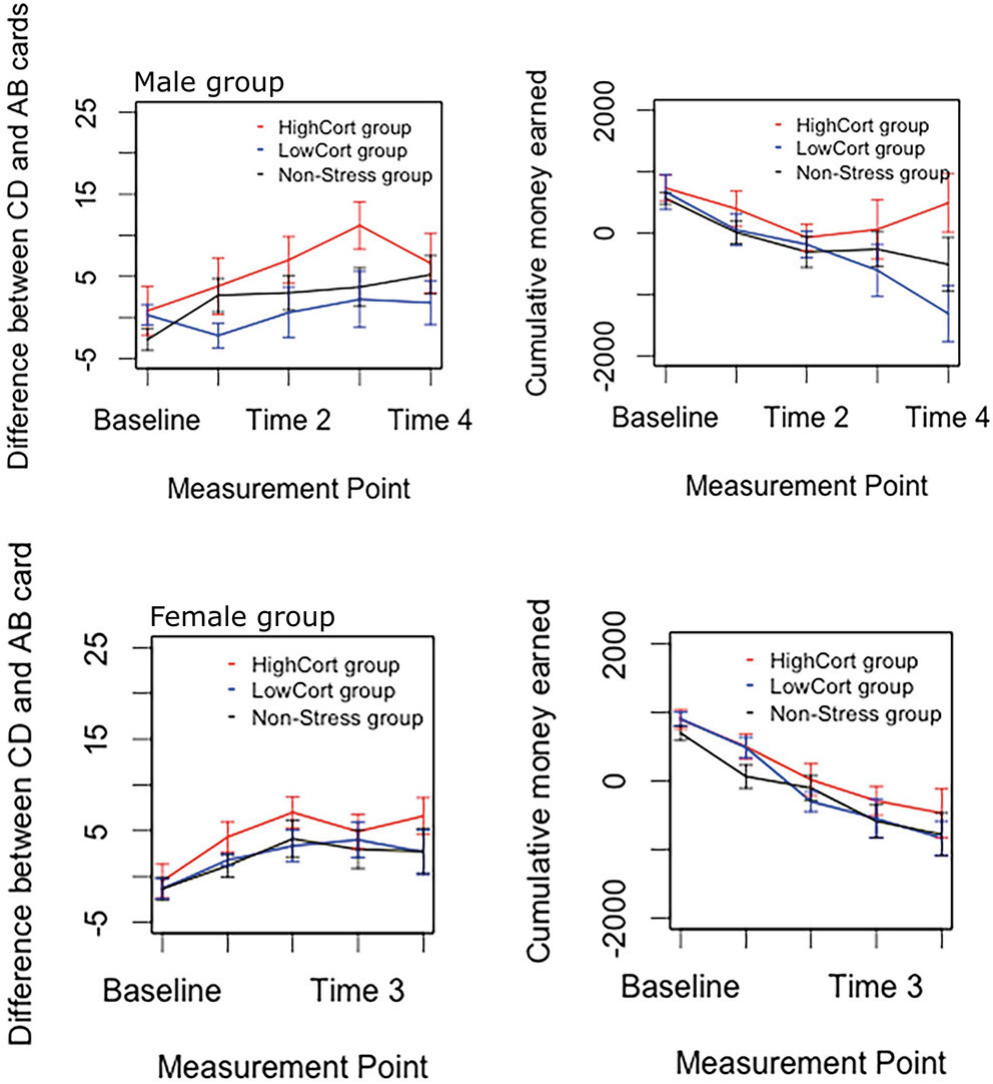
Male and female IGT performance, for each of the three groups separately. The top panel shows male IGT performance and the bottom panel female IGT performance. Error bars indicate standard error of means, with 95% confidence interval.

Analyses of the data depicted in Figure 3 indicated two findings of primary importance. First, there was a significant main effect of Group for both IGT outcome variables: CD-AB cards, *F*_(2, 360)_ = 6.49, *p* = 0.002, $\eta_{\text{p}}^{2}$ = 0.035, and cumulative money earned, *F*_(2, 360)_ = 4.91, *p* = 0.008, $\eta_{\text{p}}^{2}$ = 0.027. Second, there was no significant main effect of Sex on any IGT outcome variable, *F*s < 0.04, *p*s > 0.84, $\eta_{\text{p}}^{2}$s < 0.001.

Follow-up pairwise comparisons of the significant effect, using Tukey's HSD test, indicated that, for each outcome variable, (a) participants in the HighCort group (CD-AB cards: *M* ± *SD* = 5.17 ± 8.42; cumulative money earned: 224.05 ± 1,002.03) made significantly safer decisions than those in the LowCort group (CD-AB cards: *M* ± *SD* = 1.27 ± 6.69; cumulative money earned: −175.34 ± 1,069.77), *p* = 0.003, and 0.017, respectively; (b) participants in the HighCort group (CD-AB cards: *M* ± *SD* = 5.17 ± 8.42; cumulative money earned: 224.05 ± 1,002.03) made significantly safer decisions than those in the Non-Stress group (CD-AB cards: *M* ± *SD* = 2.15 ± 8.810; cumulative money earned: −122.87 ± 1,158.72), *p* = 0.008 and 0.015, respectively; and (c) there were no significant differences between the LowCort and Non-Stress groups, *p*s > 0.664.

Of secondary importance is that the analyses detected, for each outcome variable, a significant main effect of Block (expected for CD-AB cards because previous IGT literature suggests participants display safer behavior with increasing exposure to the task, and expected for the other two outcome variables as the amounts accumulate over time), 6.55 < *Fs* < 137.50, *p*s <0.001, 0.068 < $\eta_{\text{p}}^{2}$ < 0.604. These analyses detected no significant interaction effects, *F*s < 1.01, *p*s > 0.37, $\eta_{\text{p}}^{2}$s < 0.021.

The Supplementary Material file presents a summary of analyses describing the main effects of stress on IGT performance.

#### Risky Decision Making in the Online Chatroom

**Figure 4:**
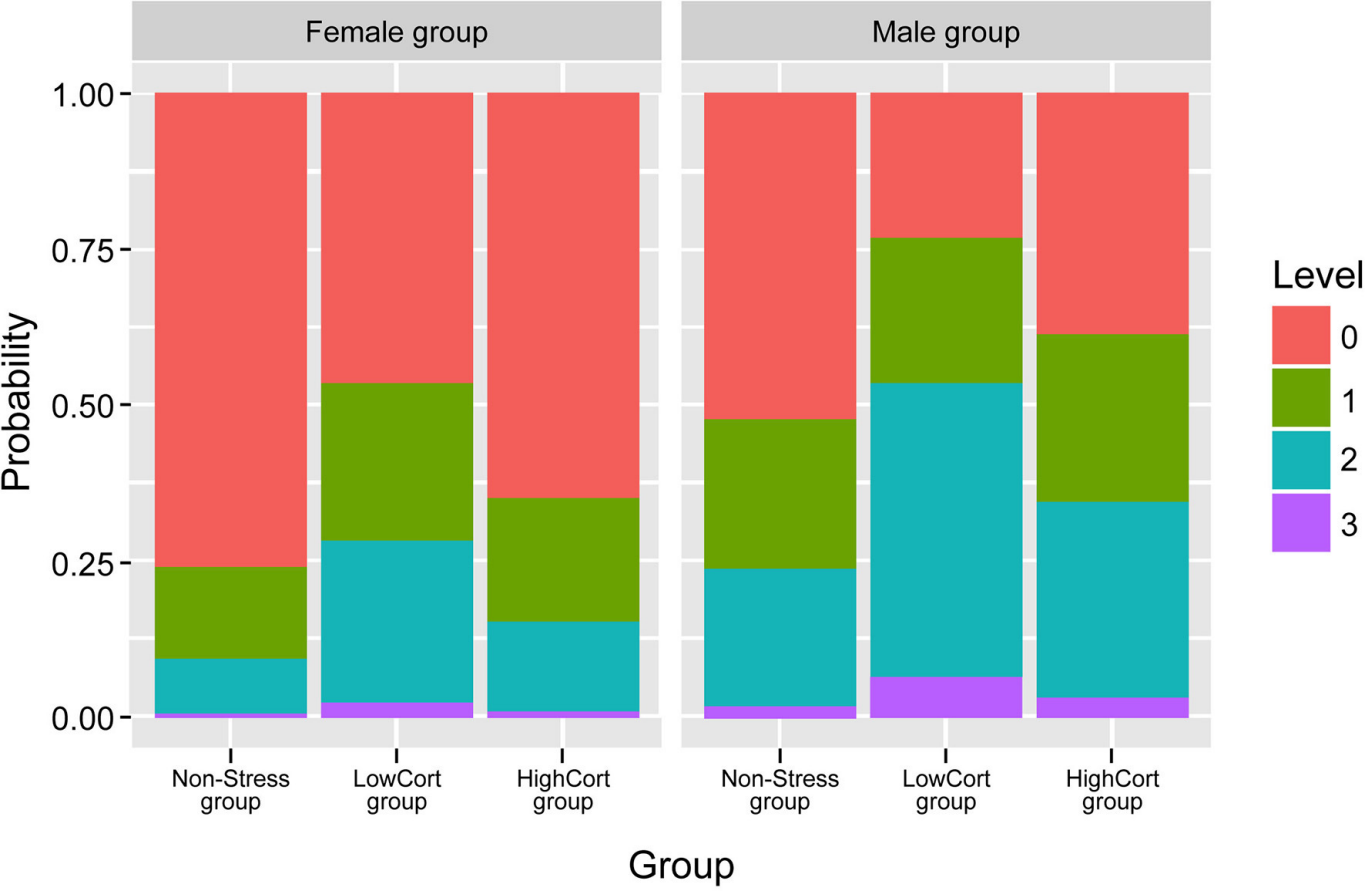
Probability of the *willingness to take a risk* in the online chatroom, for men and women separately. 3, made an offer and gave details of where to meet; 2, accepted an offer and gave details; 1, accepted or made the offer and gave limited details; 0, rejected an offer. Error bars indicate standard error of means, with 95% confidence interval.

The logistic regression model indicated that, with regard to the Group factor, participants in the LowCort group were, relative to those in the Non-Stress group, more likely to make or accept an offer to meet one of our confederates in person (in fact, the odds ratio suggests that a change from being in the Non-Stress group to being in the LowCort group means a participant was 3.6 times more likely to make that risky decision, *p* = 0.019; see Figure 4; Table 2). In contrast, being in the HighCort group vs. being in the Non-Stress group did not confer significantly higher odds of making a risky decision, *p* = 0.325. Finally, the model indicated that the odds of a woman making a risky decision was significantly lower than that of a man, *p* = 0.021, a finding consistent with the extant literature (Byrnes et al., 1999; d'Acremont and Van der Linden, 2006; Charness and Gneezy, 2012; Georgiou et al., 2018; Sidlauskaite et al., 2018).

**Table 2:** Ordinal logistic regression analysis: predicting risk decision-making by group membership (*N* = 79).

					**95% CI for** ***e***^**β**^	
**Predictor**	***Estimates***	***SE***	***p***	***e^**β**^***	**Lower**	**Upper**
Group						
LowCort vs. Non-Stress	1.28	0.55	0.019^*^	3.60	1.25	10.73
HighCort vs. Non-Stress	0.53	0.54	0.325	1.70	0.58	4.91
Sex	−1.05	0.45	0.021^*^	0.35	0.14	0.84

* *p <0.05*.

As part of the model-building process, we initially created a model that included Sex × Group interactions to determine if men and women behaved differently in the chatroom depending on their group assignment (HighCort vs. LowCort vs. Non-Stress). The predicted interaction, however, did not appear for either the LowCort × Sex (*p* = 0.875) or HighCort × Sex (*p* = 0.552) interactions. Furthermore, a model that did not include the interaction was a better fit to the data, ΔAIC = 3.46.

In summary, the model suggests that when experiencing relatively small cortisol elevations, but not when experiencing relatively greater elevations, both women and men are more likely to make a risky decision in the chatroom.

The Supplementary Material file presents a summary of analyses describing the main effects of stress on chatroom behavior.

## Discussion

The present study investigated effects of exposure to an acute psychosocial stressor, as marked by elevated cortisol levels, on risky decision making by healthy young men and women on a standardized laboratory-based measure (the Iowa Gambling Task) and in novel task that simulated a real-life social situation (an online chatroom). Chatrooms offer an excellent opportunity to study risk decision making due to the real-life, modern-day risks associated with chatroom use.

We confirmed that exposure to the stressor significantly elevated cortisol levels in participants and that, consistent with previous reports, such exposure increased salivary cortisol in men more than in women (see, e.g., Liu et al., 2017; Wolf, 2019). In our study, cortisol levels were highest during administration of the decision-making tasks (i.e., those tasks were administered between 10 and 35 mins after stressor offset, and on average peak cortisol values were reached at ~20 mins post-offset). Hence, our subsequent analyses evaluated effects of the initial wave of hypothalamic-pituitary-adrenal (HPA) axis responses to stressor exposure and not the effects of increased catecholamines associated with the relatively rapid autonomic nervous system-based arm of the stress response (i.e., the arm that typically occurs immediately after the onset of the stressor and returns to baseline about 10 mins after its cessation; Hermans et al., 2014).

We summarize the results of those subsequent analyses below, placing them in the context of our a priori predictions and the relevant literature.

Our first hypothesis was that, on both decision-making tasks, stressor-exposed participants will make more risky decisions than non-exposed participants. This hypothesis was confirmed, but only for decision making in the online chatroom. On the IGT, high-cortisol responders made significantly safer decisions than low-cortisol responders or those unexposed to the stressor, who did not detectably differ. In the chatroom, however, low-cortisol responders (but not high-cortisol responders) were, relative to unexposed participants, more likely to make a risky decision.

This disparity between results from the IGT and the chatroom may be attributed to two factors. The first factor, as Starcke and Brand (2016) reported in their meta-analysis, is that effects of stressor exposure tend to vary depending on whether the task situation is disadvantageous or not. However, whereas we found stress effects (albeit at different points on the cortisol curve) on both the disadvantageous IGT and advantageous chatroom, they found that stress affects disadvantageous but not advantageous tasks (i.e., that there were significant behavioral differences between stressed and non-stressed participants only when making a riskier decision led to fewer rewards). We did not ask our participants if they thought making a risky decision in the chatroom would be advantageous (i.e., would lead to more rewards). However, we can assume they hold this belief because Mini-K scores indicated that, on average, our participants had a slower life history strategy and the chatroom platform could provide an opportunity to meet a long-term partner. Moreover, it is possible that Starcke and Brand's (2016) proposal does not capture the nuances of all risky decision-making contexts, and that the effects of stress on some types of situations have yet to be described adequately.

The second factor concerns the characteristics of the decision-making tasks. The IGT, and other standardized laboratory-based measures of risk decision making, may not produce the same behavioral patterns as those derived from the online chatroom and other face valid tasks that attempt to simulate real-life situations. It seems plausible to argue that whereas the former tasks create contexts within which the participant risks nothing personal, the latter create contexts within which participants might perceive that their personal pride or psychological integrity are at risk. This line of argument is consistent with extant literature demonstrating the differential impact of sexual and physiological arousal on risky decision making. For instance, whereas Ariely and Loewenstein (2006) found that sexually aroused participants tended to make more risky sexual decisions, Schmidt et al. (2013) found that when physiologically aroused participants tended to make less risky sexual decisions. Although both these studies measured arousal (*via* self-report in the former case and heart-rate monitors in the latter case) and not cortisol/stress, the direction of effects they describe corresponds with our findings if one, not unreasonably, characterizes the IGT as an economic decision-making task and the online chatroom as a sexual decision-making task.

A further note in this regard is that, although the IGT's construct validity appears sound (Bechara et al., 1996), it remains unclear whether performance on the task allows one to predict real-life decision-making behavior accurately (Buelow and Suhr, 2009). Recently published studies (e.g., Shields et al., 2016; Starcke et al., 2016) provide results consistent with the proposal that standard laboratory-based decision-making tasks do not measure the same construct as tasks that mimic real life more accurately, and therefore may misrepresent the effects of stress on risk decision making (Buelow and Suhr, 2009). Such speculation and proposals can be examined by careful study and direct comparison of the two kinds of tasks. We offer the online chatroom as one platform to measure risky decision making in the hope that it allows researchers to conduct comparative studies and, more broadly, to investigate the circumstances under which individuals make risky decisions in real life.

Our second hypothesis was that, on both decision-making tasks, men will show more risk-taking behavior than women. This hypothesis was not supported: The analyses detected no sex differences in IGT performance or in online chatroom behavior. Our results are consistent with those of the Starcke and Brand's (2016) meta-analysis, which indicated that sex does not detectably moderate relations between stressor exposure and risky decision making. However, the present results stand in contrast to those from many previous studies indicating that women tend to make safer decisions where men tend to make riskier decisions (Preston et al., 2007; Lighthall et al., 2009, 2012; van den Bos et al., 2009, 2014; Mather and Lighthall, 2012; Daughters et al., 2013).

A possible explanation for this discrepancy relates to cross-study differences in the magnitude of cortisol response to psychosocial stressors. Consider the relation between cortisol response and IGT results in our study and in that of van den Bos et al. (2009), who used a near-identical procedure and a similar analytic strategy. The van den Bos et al. male high-cortisol responder group had an average baseline-to-peak elevation of about 8.5 nmol/l, whereas the analogous value for our male high-cortisol responder group was 11.87 nmol/l. Similarly, whereas van den Bos et al. reported that average absolute cortisol levels taken before and after IGT performance were ~11 and 17 nmol/l, in the current study the analogous values were 20.30 and 24.06 nmol/l. In other words, for this group of male participants the magnitude of cortisol response in our study was clearly greater, which might explain why the van den Bos et al. male high-cortisol responder group made riskier decisions than non-stressed men and in the current study, the male high-cortisol responder group made safer decisions than male low-cortisol responders or non-stressed men.

In contrast, for female high-cortisol responders values for the variables mentioned above were similar across the two studies (average baseline-to-peak elevation: van den Bos et al. ≈5 nmol/l, current study 5.7 nmol/l; average absolute cortisol levels taken before and after IGT performance, van den Bos et al. ≈10 and 16 nmol/l, current study 10.27 and 13.31 nmol/l), and in both studies female high-cortisol responders made safer decisions than non-stressed women.

In other words, across these studies men and women with relatively high salivary cortisol elevations made safer decisions than men and women not exposed to the stressor. Viewed in this light, the current data replicate a result reported by van den Bos et al. (2009) using near-identical procedures and analyses and ensuring the data were collected under similar physiological circumstances. Although the Starcke and Brand (2016) meta-analysis found that cortisol levels did not moderate the relationship between stress and decision-making, to our knowledge no study has assessed if the actual magnitudes of cortisol elevations above baseline predict risky decision making or not.

Our third hypothesis was that stressor-exposed women with relatively smaller magnitudes of cortisol elevation make safer decisions whereas those with relatively larger magnitudes make riskier decisions. This hypothesis was disconfirmed. On the IGT and in the chatroom, women (and men) with smaller cortisol elevations tended to make riskier decisions than those with larger elevations.

Again, our data are inconsistent with the findings of the Starcke and Brand (2016) meta-analysis (their review found that cortisol response did not moderate risky decision making) and with the specific pattern of behavior proposed by van den Bos et al. (2009). Whereas they found that cortisol elevations within the low-to-moderate range are associated with more or less linear increases in safe decision-making but that elevations above a certain threshold are associated with risky decision-making, we found that low-to-moderate elevations are associated with riskier decision making whereas higher elevations are associated with safer decisions. Again, this between-study discrepancy might be explained by between-study differences in magnitudes of cortisol response. For instance, the median salivary cortisol values for participants in our Stress Male and Stress Female groups were 4.875 and 1.65 nmol/l, respectively, which are higher than the 4.2 (men) and 0.8 (women) nmol/l reported by van den Bos et al. (2009).

### Limitations and Directions for Future Research

The inferences drawn from the results of this study must be tempered by the following empirical and design limitations. First, the stress induction method did not elevate cortisol levels as much in women as in men. Although this is a common finding in studies using variants of the TSST (see, e.g., Liu et al., 2017; Wolf, 2019), and is expected given sexual dimorphism in stress responses arising from the interaction of multiple neurobiological factors (e.g., different activational and organization effects of gonadal hormones on HPA-axis functioning; Bale and Epperson, 2015), this sex difference means that interpretation of findings is complicated by questions about, for instance, whether women experience as intense a stressful experience as men and how their behavior would be affected should the magnitude of cortisol response be equivalent to that of men. Hence, future studies in this field should use stress-induction methods that elevate cortisol similarly in women and men.

Second, our interpretation of the effects of elevated cortisol on the two decision-making tasks may be confounded by the fact that we did not counterbalance task presentation (i.e., for all participants, Chatroom Session 2 started 10 mins after stressor offset and the IGT started 25 mins after stressor offset). Hence, it is possible that magnitudes of cortisol elevation were different during completion of the two tasks, and that those differences might explain across-task variability in decision-making behavior. To rule out this possibility, future studies should incorporate counterbalanced task presentation.

Third, we did not measure testosterone, which previous literature indicates has important effects on decision making (particularly that made in sexualized contexts; Ronay and Von Hippel, 2010; Apicella et al., 2015; Alacreu-Crespo et al., 2019). Future studies that measure both cortisol and testosterone may be able to describe the separate and interactional effects of those hormones on risky decision making.

A fourth limitation involves our assumption of the chatroom procedure's construct and/or ecological validity—the assertion that participants believe it was a real chatroom and therefore acted in that situation as they would in a naturalistic setting. Also, it is not clear which naturalistic situations the chatroom reflects as decision-making behavior is often context-dependent. Although we can assume that the chatroom simulates an online dating context or a sexualized social situation, the study design did not include a task manipulation check and so it is difficult to know whether the assumption holds up to scrutiny. Although none of the participants betrayed any suspicion about the chatroom interactions, future studies using this task should include a formal manipulation check. They might also (a) examine ways in which the relationship status of participants might affect their decisions around taking the risk of meeting a relative stranger, and (b) consider having the participant spend more time in the chatroom so as to facilitate more interaction and allow the collection of more textual data.

### Conclusion and Significance

Understanding whether, and how, acute psychosocial stress affects risky decision making in real life has far-reaching consequences because every human will have occasion to make an important decision under pressured and anxiety-provoking circumstances. We showed that, on a standardized laboratory measure of risk decision making (the Iowa Gambling Task), both men and women characterized as low-cortisol responders (as well as those in the Non-Stress group) made significantly riskier decisions than those characterized as high-cortisol responders. Similarly, on a novel real-life valid task (an online chatroom that presented a sexually suggestive social context), both men and women characterized as low-cortisol responders (but not those characterized as high-cortisol responders) were, relative to those in the Non-Stress group, significantly more likely to make risky decisions. Together, these results suggest a dose response between stressor-induced cortisol elevations and risky decision making, and that this relationship might be observed in both men and women across different task situations. Although our results are generally consistent with trends in the literature, the novelty of our study is the demonstration that risky decision making under stressful conditions is better predicted by the participant's magnitude of cortisol elevation than their sex or by the type of decision-making task.

## Data Availability Statement

The raw data supporting the conclusions of this article will be made available by the authors, without undue reservation.

## Ethics Statement

The studies involving human participants were reviewed and approved by University of Cape Town, Faculty of Health Sciences, Human Research Ethics Committee. The patients/participants provided their written informed consent to participate in this study.

## Author Contributions

AD, DS, RH, TS, LA, AO'N, WJ, and KT contributed to the design and implementation of the research. AD and KT contributed to the analysis of the results and to the writing of the manuscript. All authors discussed the results and commented on the manuscript. All authors contributed to the article and approved the submitted version.

## Funding

This research was supported by the Oppenheimer Memorial Trust and the National Research Foundation of South Africa.

## Conflict of Interest

The authors declare that the research was conducted in the absence of any commercial or financial relationships that could be construed as a potential conflict of interest.

## Publisher's Note

All claims expressed in this article are solely those of the authors and do not necessarily represent those of their affiliated organizations, or those of the publisher, the editors and the reviewers. Any product that may be evaluated in this article, or claim that may be made by its manufacturer, is not guaranteed or endorsed by the publisher.

## Footnotes

^1^Broadly speaking, we follow Figueredo and Jacobs (2010, p. 9) in noting that “Behavioral ecology and Economics define risk mathematically as increased variance in outcomes. Outcomes may be distributed symmetrically about a mean. Hence it may be adaptive, under certain circumstances, to take so-defined risks. With a mean expected outcome or *payoff* of zero, there is no a priori theoretical reason to avoid taking such risks consistently because there is no consistently expected *loss* associated with outcomes”.

^2^Difference-score outcome variables (CORTΔ1/CORTΔ2/CORTΔ3/CORTΔ4) were calculated by subtracting the CORT_B_ value from each cortisol value at the other measurement points (i.e., CORT1, CORT2, CORT3, CORT4).
