# Supplementary material for: Risky Decision Making Under Stressful Conditions: Men and Women With Smaller Cortisol Elevations Make Riskier Social and Economic Decisions
Source: Front Psychol. 2022 Feb 4;13:810031. doi: 10.3389/fpsyg.2022.810031 (PMC8854750; doi:10.3389/fpsyg.2022.810031)
Supplement: Supplementary file 1 [file Data_Sheet_1.docx]

**Supplementary material**

**S.1. Personality Types for Chatroom Confederates**

**1.1. Female participant**

**ThomasTheTank Engine**

Chatter is forward and engaging if the participant is not. If the participant plays a passive role, takes a lead in the conversation, initially vague but a little flirty:

- Looks: Muscle build, tall, blonde hair, blue eyes
- Basic interests: Sports, gym, partying, hobbies, - this individual is a popular, good looking male. He likes to party and socialize.
- Studying BComm

**Packetman**

Chatter is equally engaging but not as flirty. More of the guy next door – but also knows how to have fun. Sporty, athletic, out-doors type.

- Looks: tanned and athletic, all about being able to get out in nature. Possible leading sports – hiking, running, mountain biking
- Basic interests: wanting to be a clinical psychologist, interest in helping people – good and wholesome.
- Studying Psychology

**CoolGirl**

Takes a passive role in the chat and mostly takes a back seat and chips in occasionally to remind the participant she is not the only female in the chatroom.

- Quite academic and hardworking, studying Science
- Not too sure about her plans for the future, just studying for now
- Doesn’t go out much or socialize, doesn’t really like going to the beach or being outdoors much
- Makes inappropriate remarks at times. An example being: No need to compensate for a lack of size in the pants.

**1.2. Male participant**

**Commgirl**

Chatter is forward and engaging if the participant is not. If the participant plays a passive role, takes a lead the conversation, initially vague but a little flirty:

- Looks: Slim, tall, blonde, blue eyes
- Basic interests: Sports, partying, hobbies, - this individual is a little more of a party girl, likes to let her hair down and go out on the weekends
- Studying a BComm

**2_cool**

Chatter is equally engaging but not as flirty. More of the girl next door – but also knows how to have fun. Sporty, athletic, outdoors type.

- Looks: tanned and athletic, all about being able to get out in nature. Possible leading sports – horse riding, gymnastics
- Basic interests: wanting to be a clinical psychologist, interest in helping people – good and wholesome. Studying Psychology

**JP13**

Passive role in the chat – takes a back seat and pipes in occasionally to remind the participant he is not the only male.

- Quite academic and hardworking, studying engineering
- Enjoys engineering, but is not sure about his plans for the future
- Doesn’t go out much or socialize, enjoys computer games, doesn’t really like going to the beach or being outdoors much
- Makes inappropriate remarks at times. An example being: love the shirt skirts and tight jeans girls are wearing on campus these days. Gets me excited.

**S.2. Relationship between cortisol increase and Performance on Decision-Making Tasks**

We wanted to investigate the relationship between the increase in salivary cortisol levels over baseline and the performance on the decision-making tasks to investigate whether the increase in cortisol is associated with decision-making outcomes (so that we can be confident that the results of the decision-making tasks are because of cortisol increases). On average, cortisol levels were highest at CORT_2_, therefore the difference between this value and the CORT_B_ value (CORT∆2) was used to investigate this question.

**2.1. Risky decision making on the IGT**

For each outcome variables of the IGT (i.e., CD-AB Cards score, cumulative money earned), we conducted linear regression models with CORT∆2 as the single predictor and the score at IGT_5_ for each of the IGT outcome variables. The linear regression models indicated that there was a significant association between cortisol increase and cumulative money earned, *F*(1, 76) = 5.56, *β* = 69.40, *p* = .021. There was not a significant association between CD-AB cards and cortisol increase, *F*(1, 76) = 0.97, *β* = 0.187, *p* = .327.

**2.2. Risky decision making** **in the online chatroom**

Regarding performance on the online chatroom, we conducted an ordinal logistic regression model to examine whether the increase in cortisol levels over baseline was associated with willingness to take a risk. There was not a statistically significant association between cortisol increase and willingness to take a risk, *p* = .763, odds ratio = 1.01 (95% CI 0.94-1.09).

**S.3. Performance on Decision-Making Tasks by Stress vs. Non-Stress Groups**

We wanted to investigate performance on the decision-making tasks by the Stress vs. Non-Stress Groups (i.e. before the Stress Group was divided into the HighCort and LowCort groups) to investigate the main effect of Experimental Condition on decision-making outcomes.

**3.1. Risky decision making on the IGT**

For each outcome variables of the IGT (i.e., CD-AB Cards score, cumulative money earned), we conducted a 2 (Experimental Condition: Stress vs. Non-Stress) x 5 (Block: IGT_1_, IGT_2_, IGT_3_, IGT_4_, IGT_5_) x 2 (Sex: Women vs. Men) repeated-measures ANOVA.

Analyses of the data indicated that there was no significant main effect of Experimental Condition for the two IGT outcome variables: CD-AB cards, *F*(1, 370) = 1.81, *p* = .180, *η*_p_^2^ = .005 and cumulative money earned, *F*(1, 370) = 2.16, *p* = .143, *η*_p_^2^ = .006. Furthermore, there was no significant main effect of Sex on any of the the IGT outcome variables, *F*s < 0.07, *p*s > .790, *η*_p_^2^s < .001.

Lastly, the analyses detected, for each of the two outcome variables, a significant main effect of Block, 6.44 < *Fs* < 132.94, *p*s < .001, .065 < *η*_p_^2^ < 1.74. These analyses detected no significant interaction effects, *F*s < .84, *p*s > .50, *η*_p_^2^s < .009.

**3.2. Risky decision making** **in the online chatroom**

Regarding performance on the online chatroom, we conducted an ordinal logistic regression model to examine whether Experimental Condition (Stress, Non-Stress) and Sex (female, male) predicted willingness to take a risk. The logistic regression model indicated that, with regard to the experimental manipulation, participants in the Stress group were, relative to those in the Non-Stress group, more likely to make or accept an offer to meet one of our confederates in person (in fact, the odds ratio suggests that a change from being in the Non-Stress group to being in the Stress group means a participant was 2.5 times more likely to make that risky decision, *p* = .047 ; see Table S1). Finally, the model indicated that the odds of a woman making a risky decision was significantly lower than that of a man, *p* = .024.

As part of the model-building process, we initially created a model that included Sex x Experimental Condition interactions to determine if men and women behaved differently in the chatroom depending on whether they were exposed to the stressor or not (Stress vs Non-Stress). The predicted interaction, however, did not appear for the Experimental Condition x Sex (*p* = .844) interaction. Furthermore, a model that did not include the interaction was a better fit to the data, ΔAIC = 1.96.

In summary, the model suggests that participants exposed to the stressor are more likely to make a risky decision in the chatroom.

Table S1.

*Ordinal Logistic Regression Analysis: Predicting risk decision-making by group membership (N = 79)*

|  |  |  |  |  | 95% CI for *e^β^* | |
| --- | --- | --- | --- | --- | --- | --- |
| Predictor | *Estimates* | *SE* | *p* | *e^β^* | Lower | Upper |
| Experimental Condition | 0.89 | 0.45 | .047* | 2.45 | 1.02 | 6.03 |
| Sex | 1.01 | 0.45 | .024* | 2.76 | 1.16 | 6.79 |

**p* < .05
